# Value of computed tomography for evaluating the subglottis in laryngeal and hypopharyngeal squamous cell carcinoma

**DOI:** 10.1590/S1516-31802007000200002

**Published:** 2007-03-04

**Authors:** Ricardo Pires de Souza, Nestor de Barros, Ademar José de Oliveira Paes, Olger de Souza Tornin, Abrão Rapoport, Giovanni Guido Cerri

**Keywords:** Cancer, Neck, Radiology, Larynx, Hypopharynx, Câncer, Pescoço, Radiologia, Laringe, Hipofaringe

## Abstract

**CONTEXT AND OBJECTIVE:**

Subglottic involvement in squamous cell carcinoma is a determining factor for contraindicating conservative partial surgery. The subglottis is easily identified by axial computed tomography sections. The present study aimed to evaluate the occurrence of false-negative and false-positive results, and the overall accuracy of staging by computed tomography, in order to detect the involvement of the subglottic laryngeal compartment, in cases of laryngeal and hypopharyngeal squamous cell carcinoma.

**DESIGN AND SETTING:**

Retrospective, non-randomized study of patients treated at Hospital Heliópolis, São Paulo, Brazil.

**METHODS:**

Computed tomography scans were performed on third-generation equipment with 5-mm slice thickness. Afterwards, all patients underwent surgical and anatomopathological examinations as the gold standard procedures.

**RESULTS:**

Among 60 patients, 14 were diagnosed with subglottic extension by surgical and histopathological examination. There were three false-negative and no false-positive results from computed tomography scans. The sensitivity and negative predictive value were 100.0%. Accuracy was 95.0%, specificity was 93.5% and positive predictive value was 82.4%.

**CONCLUSIONS:**

Computed tomography could serve as a powerful auxiliary method for staging laryngeal and hypopharyngeal cancer. However, precautions should be taken in analyzing computed tomography scan data, because vegetating lesions may also be projected into the subglottic compartment, without real involvement of the subglottis, which may cause a false-positive result.

## RESUMO

**CONTEXTO E OBJETIVO:**

O envolvimento subglótico, em casos de carcinoma espinocelular, é fator determinante na contra-indicação da cirurgia parcial conservadora. A subglote pode ser facilmente identificada no cortes axiais da tomografia computadorizada. O presente estudo tem por objetivos avaliar a ocorrência de resultados falsos negativos, falsos positivos e a eficácia global do estadiamento por tomografia computadorizada na detecção do envolvimento do compartimento subglótico da laringe, em casos de carcinoma espinocelular de laringe e hipofaringe.

**TIPO DE ESTUDO E LOCAL:**

Retrospectivo não randomizado, realizado no Hospital Heliópolis, São Paulo, Brasil.

**MÉTODOS:**

Os exames de tomografia computadorizada foram realizados em equipamentos de terceira geração, com cortes com espessura de 5 mm. Todos os pacientes foram posteriormente submetidos a cirurgia com comprovação anatomopatológica, sendo este o padrão ouro.

**RESULTADOS:**

Dentre os 60 pacientes, o envolvimento subglótico ocorreu em 14, conforme observado no achado cirúrgico e ou anatomopatológico. A avaliação pela tomografia computadorizada resultou em três casos falsos positivos. Não ocorreram resultados falsos negativos. A tomografia computadorizada apresentou 100,0% de sensibilidade, 100,0% de valor preditivo negativo e 95,0% de eficácia global. A especificidade foi de 93,5% e o valor preditivo positivo de 82,4%.

**CONCLUSÕES:**

Consideramos que a TC pode ser um poderoso instrumento auxiliar no estadiamento do câncer de laringe e de hipofaringe, devendo-se ter precaução com lesões vegetantes que se projetam para a subglote sem seu verdadeiro envolvimento.

## INTRODUCTION

Squamous cell carcinoma (SCC) consists of epithelial tumors of mostly mucosal origin. SCC is the most common form of malignant tumor in the pharynx and hypopharynx, accounting for approximately 90% of the malignant tumors in these areas.^1^ It is potentially curable by treatment with surgery and radiotherapy, and these constitute the main therapeutic methods.^2^

The possible surgical treatments for laryngeal and hypopharyngeal carcinoma include total laryngectomy, with lost of the voice, and partial laryngectomy, which preserves phonatory functions.^3^

Indication of procedures that conserve the voice, such as radiotherapy and partial laryngectomy, depends on preoperative staging of the lesion such that its extent is not underestimated^4^ and the principle of oncological radicality is consequently not disregarded.

Diagnostic and clinical staging of laryngeal and hypopharyngeal carcinoma are usually performed by clinical examination, indirect laryngoscopy, direct laryngoscopy and biopsy with histopathological examination.^5^

Laryngoscopic examination allows a close approach to the mucosal areas of the larynx and hypopharynx, as well as evaluation of the lesion extent in these areas.^6^ However, subglottic involvement may be an exception, since this is difficult or impossible to evaluate by laryngoscopic examination, especially in cases of large, bulky tumors or when the patient’s respiratory state does not allow for such examination.^7^

Subglottic involvement is a determining factor for contraindicating conservative partial surgery.^8^ Indication of radiotherapy in such cases is controversial.^9-11^

**Figure 1:**
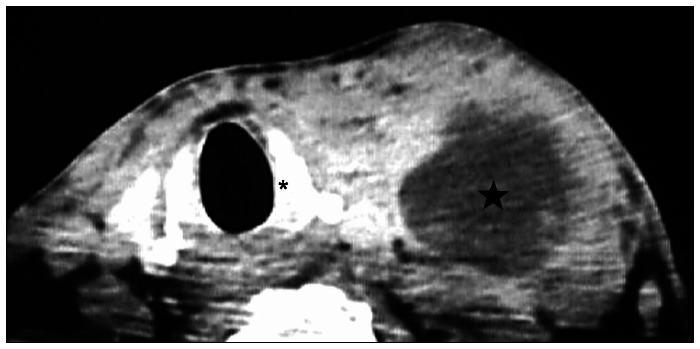
Axial computed tomography scan at the cricoid cartilage (*) level. Normal appearance of subglottis. True-negative example. The scan shows left lymph node enlargement (star).

The subglottis is easily assessed by axial computed tomography (CT) at the level of the cricoid ring.^12^ The subglottic mucosa is closely connected to the surface of the internal cricoid cartilage, and any thickening or irregularity at this level is usually not identified^13-15^ (Figure 1).

## OBJECTIVE

The present study aimed to evaluate the occurrence of false-negative and false-positive results and the accuracy of staging by CT for detecting involvement of the subglottic laryngeal compartment, in cases of laryngeal and hypopharyngeal squamous cell carcinoma.

## MATERIAL AND METHODS

Sixty patients with laryngeal and hypopharyngeal squamous cell carcinoma were evaluated and treated at the Head and Neck Surgery Ser-vice of Hospital Heliópolis (Hosphel), São Paulo, Brazil, from 1991 to 1996.

Fifty-four patients (90.0%) were male and six were female (10.0%), with ages ranging from 37 to 76 years.

Fifty-four patients (91.6%) had a history of smoking and 48 (80.0%) had a history of alcohol consumption.

The most common initial complaint was hoarseness (n = 35/60 or 58.3%), followed by odynophagia (n = 12/60 or 20.0%), neck nodule (n = 7/60 or 11.6%), sore throat (n = 4/60 or 6.6%) and foreign body sensation in the throat (n = 2/60 or 3.3%).

The time elapsed between symptom appearance and the clinical diagnosis of cancer ranged from one to 48 months. For 31 patients (51.6%), this time was less than or equal to six months, and for 53 patients (88.3%) it was less than or equal to 12 months.

All patients underwent indirect laryngoscopy and 52 underwent direct laryngoscopy.

Forty-three patients underwent total laryngectomy and seventeen had some type of conservative surgery.

Among 17 tumors (28.3%) originating from the hypopharynx, 16 (26.6%) were in the pyriform sinus and one (1.6%) in the posterior wall. There were 43 laryngeal tumors (71.7%), and these were distributed in the following order: vocal cords: n = 14 (23.3%); epiglottis: n = 10 (16.6%); ventricle: n = 9 (15.0%); ventricular band: n = 6 (10.0%); and aryepiglottic fold: n = 4 (6.6%).

The local staging or T parameter for the tumors, according to the TNM scale of the Union Internationale Contre le Cancer (UICC) classification (1992), was distributed thus: Tx: n = 3 (5.0%); T1a: n = 3 (5.0%); T1b: n = 1 (1.6%); T2: n = 15 (25.0%); T3: n = 28 (46.6%); and T4: n = 10 (16.6%).

None of the patients had previously undergone any kind of treatment.

All the CT scans were performed on third-generation equipment, with 5-mm slice thickness and identical increments, guided by digital profile radiography. Intravenous contrast medium (iohexol 300 mg/ml) was administered to 58 patients (96.7%).

Involvement of the subglottic compartment was characterized when any irregularity or thickening of the soft tissue along the inner margin of the cricoid cartilage was identified on the CT scan.

The results from the CT scan examinations were compared with the findings from the direct and indirect laryngoscopy examinations, and with the surgical findings and/or histopathological examinations. The surgical and histopathological findings represented the gold standard.

## RESULTS

**Figure 2:**
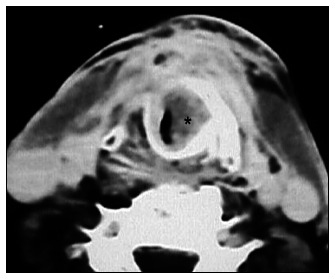
Axial computed tomography scan. Left vocal cord tumor (*). True-positive case.

Intraoperative macroscopic evaluation and/or histopathological examination on the 60 surgical specimens showed subglottic involvement in 14 cases (Figure 2) out of this series (23.3%).

Among these 60 cases in the series, indirect laryngoscopy was unable to evaluate the subglottic compartment in 26 cases (43.3%).

Out of the 52 cases (86.6%) in which direct laryngoscopy was performed, this method was unable to evaluate the subglottis in 11 cases (21.2%).

Evaluation of the subglottic compartment by CT yielded the results presented in Table 1 and the sensitivity measurement calculations (specificity, positive predictive value, negative predictive value, accuracy) in Table 2.

**Table 1:** Results from evaluation of the subglottic compartment by computed tomography

Cases	n	%
True-negative	43	71.7
True-positive	14	23.3
False-negative	0	0
False-positive	3	5.0

Confidence interval (95.0%) = 0.728-1.012.

**Table 2:** Sensitivity, specificity, positive predictive value, negative predictive value and accuracy of the computed tomography findings on subglottic involvement

	%
Sensitivity	100.0
Specificity	93.5
Positive predictive value	82.4
Negative predictive value	100.0
Accuracy	95.0

**Figure 3:**
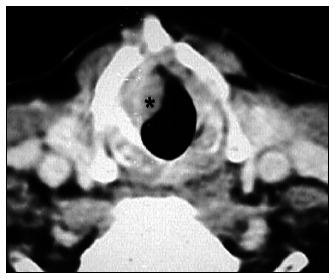
Axial computed tomography scan. Vegetative lesion (*) extending inferiorly and simulating glottis involvement. False-positive example.

**Figure 4:**
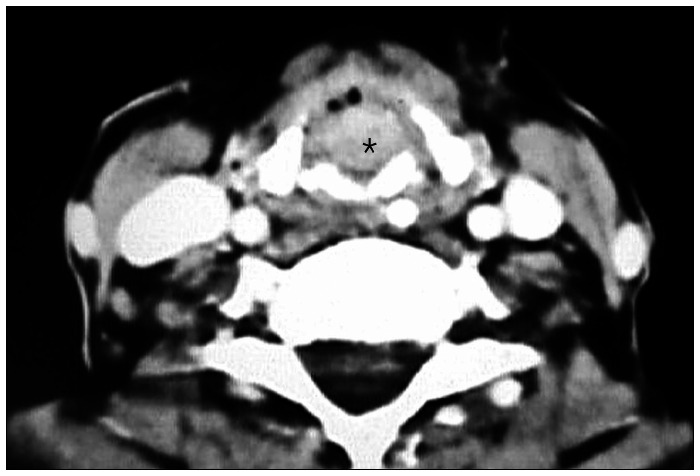
Axial computed tomography scan. Tumor (*) affecting the larynx at the glottis level.

The three false-positive cases (5.0%) found by CT in the subglottic compartment evaluation resulted from bulky polyp lesions that were projected into the inner subglottis compartment, without real involvement (Figures 3 and 4).

## DISCUSSION

Laryngeal and hypopharyngeal SCC represent more than 90.0% of the malignant tumors in these regions. SCC usually progresses slowly and more frequently affects men, particularly in their sixth and seventh decades of life. It has been shown to present its highest incidence among smokers and individuals with high alcohol consumption. In our sample, nine times more men were affected than women, eleven times more smokers than non-smokers and four times more consumers of alcohol than non-consumers. The peak incidence of the disease (n = 23/60 or 38.3%) occurred within the age range from 51 to 60 years old.

SCC typically begins in the inner laryngeal and hypopharyngeal surfaces and presents in three different types: infiltrative, bulky or mixed. A local dissemination pattern for SCC can be found on mucosa surfaces or when there is deep invasion of structures in these organs, with consequent submucosal extension.

It is a potentially curable tumor. The survival rates depend on early diagnosis and adequate treatment for each situation.

The subglottic larynx or subglottis marks the transition from stratified squamous epithelium to respiratory epithelium. It consists of a small round segment of the airway that is situated immediately under the vocal cords and above the trachea. Although primary carcinoma in the subglottis is quite rare, secondary invasion by carcinoma that began in other adjacent areas is frequent.

The diagnosis of SCC usually starts to emerge with the appearance of its symptoms: typically hoarseness caused by vocal cord involvement, or pain on deglutition (odynophagia), in cases of hypopharyngeal tumors. Sometimes, the primary tumor remains clinically silent and the initial manifestation is the appearance of a cervical nodule that represents metastasis to lymph nodes. The time elapsed between symptom appearance and making the definitive diagnosis is fundamental in determining the extent of tumor spreading.^16,17^

In addition to offering a means of obtaining material for histopathological confirmation of the tumor by biopsy, direct or indirect laryngoscopy should be capable of determining the diagnosis and precisely defining the local extent of the tumor. Nonetheless, laryngoscopy does not allow evaluation of the extent and depth of tumor invasion into the submucosa. It has to be considered that cricoid cartilage involvement, or invasion of deep tissues in the neck will necessitate total laryngectomy.^18-20^

It is difficult or impossible for laryngoscopic examination to provide information about the subglottis and the deep structures of the larynx and hypopharynx. However, there is a great need to identify tumor extent in a very precise manner. Consequently, over the course of time, imaging examinations were added to the large number of auxiliary methods for tumor staging. CT scans were thus brought in as an auxiliary method for laryngeal and hypopharyngeal tumor staging, in primary tumor evaluations and for attempting to identify and characterize metastatic cervical lymph nodes.

Not all cases of laryngeal and hypopharyngeal SCC have an indication for CT scans. In cases with early identification of small and superficial lesions that are clearly demonstrated by clinical examination and in which palpation is enough for defining the procedures to adopt, imaging examinations are not necessary.^21^

In the present study, the subglottis could be evaluated by axial CT scans obtained at cricoid ring level, in each of the 60 cases in this series. In accordance with this criterion, 17 cases in our sample were identified as positive, although three of them were shown to be false-positive by histopathological analysis. All 14 cases in which there was subglottic involvement were correctly detected.

Although there are some case reports on false-negative CT scans in the literature, such as Grandjean et al. (1993),^22^ other studies by Mafee et al. (1983),^23^ García-Alonso et al. (1992)^24^ and Kazkayasi et al. (1995)^25^ presented absence of false-negative findings. Like these authors, we did not find any false-negative results in our series of 60 patients. All 14 cases of subglottic involvement were correctly detected by CT scans, thus giving a negative predictive value of 100%. In our series, three false-positive results were found, caused by bulky lesions originating in the vocal cords that extended inferiorly and projected into the inner margin of the subglottis, without having any real involvement with this compartment. Two of these cases underwent conservative surgery after intraoperative evaluation. The third case underwent simple total laryngectomy. These false-positive results gave rise to a positive predictive value of 82.4%.

Conjointly with the absence of false-negative results, the three false-positive cases in this series resulted in accuracy of 95.0% in the subglottic evaluation of laryngeal and hypopharyngeal SCC by CT scans.

## CONCLUSIONS

According to these results, we conclude that computed tomography could serve as a powerful auxiliary method for staging laryngeal and hypopharyngeal cancer. However, precautions should be taken in analyzing CT scan data, because vegetating lesions may also be projected into the inner subglottis compartment, without its real involvement, which may cause a false-positive result.
